# Gastrin and Gastric Cancer

**DOI:** 10.3389/fendo.2017.00001

**Published:** 2017-01-17

**Authors:** Helge L. Waldum, Liv Sagatun, Patricia Mjønes

**Affiliations:** ^1^Department of Gastroenterology and Hepatology, St Olav’s Hospital, Trondheim, Norway; ^2^Department of Cancer Research and Molecular Medicine, Faculty of Medicine, Norwegian University of Science and Technology, Trondheim, Norway; ^3^Department of Pathology, St Olav’s Hospital, Trondheim, Norway; ^4^Department of Laboratory Medicine, Children and Women’s Health, Norwegian University of Science and Technology, Trondheim, Norway

**Keywords:** carcinogenesis, classification of cancer, gastric cancer, gastrin, hormones, neuroendocrine neoplasia

## Abstract

Gastric cancer although occurring in reduced frequency is still an important disease, partly because of the bad prognosis when occurring in western countries. This decline in occurrence may mainly be due to the reduced prevalence of *Helicobacter pylori* (Hp) infection, which is the most important cause of gastric cancer. There exist many different pathological classifications of gastric carcinomas, but the most useful seems to be the one by Lauren into intestinal and diffuse types since these types seldom transform into the other and also have different epidemiology. During the nearly 30 years that have passed since the groundbreaking description of Hp as the cause of gastritis and gastric cancer, a continuous search for the mechanism by which Hp infection causes gastric cancer has been done. Interestingly, it is mainly atrophic gastritis of the oxyntic mucosa that predisposes to gastric cancer possibly by inducing hypoacidity and hypergastrinemia. There are many arguments in favor of an important role of gastrin and its target cell, the enterochromaffin-like cell, in gastric carcinogenesis. The role of gastrin in gastric carcinogenesis implies caution in the long-term treatment with inhibitors of gastric acid secretion inducing secondary hypergastrinemia, in a common disease like gastroesophageal reflux disease.

Gastric cancer has been one of the most prevalent cancers and combined with a high mortality, gastric cancer has been among the leading causes of cancer death. However, there has been a continuous decline in the prevalence of gastric cancers during the last decades. Unfortunately, in spite of widespread use of upper gastrointestinal endoscopy, the prognosis in patients affected in the western world has not been much improved. This is in contrast to Japan where gastric cancer often is diagnosed at an early phase allowing removal and a better prognosis (1, 2). This is partly due to special screening programs in Japan having a high prevalence of gastric cancer (1, 2), resulting in diagnoses at a curable stage. In most western countries, the prevalence is much lower (3), and therefore, no such program has been implemented. The cause of the geographical differences in the prevalence of gastric cancer most probably is increased prevalence of *Helicobacter pylori* (Hp) in East Asia as well as a higher frequency of atrophic oxyntic gastritis (4). The prognosis of gastric cancer is better in patients from East Asia even when living in the west possibly due to less aggressive biology (5). In this review, we will focus on the role of gastrin in the etiology of gastric cancer and at the same time give an explanation of the decline in frequency. We will only cover cancers originating from epithelial cells (carcinomas) and will not discuss the importance of Epstein–Barr virus that plays a role neither in gastric carcinogenesis (6) nor in human papilloma virus, which has a less established impact (7).

## The Gastric Mucosa

The mucosa of the stomach has traditionally been divided into three parts: the cardiac, the oxyntic, and the antral mucosa. During the last decades, it has, however, been discussed whether the cardiac mucosa occurs normally or represents metaplastic mucosa (8, 9). In the oxyntic mucosa, the highly specialized glands contain the acid-producing parietal cell, the pepsinogen-producing chief cell, and the regulatory, histamine-producing [enterochromaffin-like (ECL)] cell, which are specific for the oxyntic glands. These cells are not found in the antral glands where instead the gastrin-producing G-cell is localized. Previously, a sharp border between the oxyntic and antral mucosa was presumed, but recent work has shown that there is overlap with oxyntic glandular elements occurring in the antral mucosa (10). Nevertheless, taking into consideration the differences between the oxyntic and the antral mucosa, it should be obvious that gastric carcinomas should be classified anatomically according to mucosa of origin and not as presently only into cardiac and distal carcinomas with the latter comprising both oxyntic and antral starting point.

## Embryology of the Gastric Mucosa

The gastrointestinal tract is derived from the endoderm. Stem cells located at the neck of the glands divide and differentiate into specialized cells while moving into the crypts of the glands (parietal and chief cells) or to the surface becoming specialized cells producing mucus and bicarbonate, which make the gastric mucosa like the mucosa of the duodenal bulb, able to resist the highly acidic and proteolytic gastric juice. There are many regulatory neuroendocrine (NE) cells in the gastric mucosa. The NE cells in man were previously claimed not to divide (11) in contrast to similar cells in rodents (12, 13). Now it is, however, established that NE cells also in man do divide as shown for the β-cell (14) and indirectly for the gastric ECL cell by the selective and concentration-dependent trophic effect by gastrin (15). In the gastric mucosa, the ability to self-replicate is unique to the ECL cell and probably the other NE cells and in contrast to other mucosal cells that are formed by differentiation of cells originating from stem cells. Nevertheless, studies have indicated that also the NE cell originate from a common stem cell (16, 17), and thus not coming from the neural crest as proposed by Pearse and Polak (18) based on the similarities between NE cells at different locations and neural cells. Although there seems to be rather firm evidence for stem cell origin of NE cells in the intestine and the antrum (16, 19), this has not been convincingly shown for NE cells in the oxyntic mucosa.

## Properties of NE Cells

Whatever the embryology, the NE cells have a unique position among the mucosal cells in their ability to divide. Moreover, they produce signal substances that affect the function of neighboring cells. The signal substances are delivered *via* a paracrine route or *via* synaptic-like transmission from neuron-resembling projections (20, 21) or reaching cells *via* the blood working as hormones. The NE cells have secretory granules that may be detected by the general markers belonging to the chromogranins (22), and by cell-specific signal substances such as gastrin, ghrelin, somatostatin, enteroglucagon, and many others. Interestingly from an embryological point of view, the synaptic vesicle marker synaptophysin (23) is found in NE cells in common with neurons. In the gastrointestinal tract, there are about 30–40 different NE cells producing separate signal substances. Although the different types of NE cells look quite similar, most of the NE tumors originate from only a few of them, typically the ECL cell in the stomach and the enterochromaffin (EC) cell in the gut. The reason for this discrepancy in tumourigenecity is not known, but could be related to differences in growth regulation as well as to different signal substances with different effect on surrounding tissue. Thus, not only the function (24) but also the proliferation (25) of the ECL cell in the stomach is regulated by the hormone gastrin. Gastrin is often elevated in gastric hypoacidity either due to atrophic gastritis (26) or drug treatment (27). Moreover, the signal substance of the ECL cell is histamine having profound vascular effects, which presumably may favor invasion and spread. In the small intestine, the serotonin-producing EC cell is the principal cell of origin of NE tumors. The regulation of the EC cell function and proliferation is mainly unknown, but it is possible that the serotonin production *via* its vascular effects is the cause of invasion and spread at an early phase, which is in many ways a hallmark of this tumor. Although the NE cells do proliferate, they do so very slowly which was the background for the dispute whether they divide or not (11, 28). The slow proliferation of normal NE cells is reflected in the relatively benign course of NE tumors where the patient may live asymptomatically for more than a decade with such a tumor or the first metastasis may manifest itself after more than two decades.

## Predisposing Conditions for Gastric Cancer

Gastritis with metaplasia has for long been known to predispose to gastric cancer (29). In fact, it was shown that gastric cancer virtually only occurred in stomachs with gastritis (29). When Hp was shown to be the cause of most types of gastritis (30), it was natural to examine the role of Hp in gastric cancer, and it was soon shown to be the most important etiological factor of this malignancy (31). Very recently, a meta-analysis showed that Hp eradication reduced the risk of gastric cancer (32). However, also the other type of gastritis, the so-called autoimmune gastritis affecting only the oxyntic mucosa predisposes to gastric cancer (33). Thus, it seems that the gastritis and not Hp itself is the carcinogenic mechanism. Nevertheless, throughout the 25 years since the description of Hp as the cause of gastritis (30) and gastric carcinoma (31), there have been numerous studies on the mechanism by which Hp induces gastric carcinoma, all of them without success. When Hp gastritis is confined to the antral mucosa, it predisposes to duodenal ulcer (34), but not to gastric carcinoma. On the contrary, it is well known that duodenal ulcer in a way protects against development of gastric carcinoma (35). The risk of gastric carcinoma increases only when the gastritis affects the oxyntic mucosa (36) and mainly when oxyntic atrophy has developed (36). The presence of oxyntic gastric atrophy may indirectly be assessed by low serum pepsinogen I (37) and even better when combined with determination of gastrin (38). In atrophic gastritis, intestinal metaplasia may with time develop (39). Intestinal metaplasia has been associated with an additional cancer risk but could also just reflect long-standing oxyntic atrophy as patients with intestinal metaplasia are older than those with oxyntic atrophy without intestinal metaplasia (39, 40). Anyhow, the sequence in gastric carcinogenesis postulated by Correa where the mucosa goes through phases of gastritis, atrophic gastritis, intestinal metaplasia, dysplasia of different degrees, and finally neoplasia has had great impact for a long time (41). However, in a recent Swedish study, there was little difference in risk of gastric cancer between patients with atrophic gastritis and those with intestinal metaplasia (42). As in other parts of the gastrointestinal tract, polyps in the stomach may predispose to gastric carcinomas, although the polyp carcinoma sequence in the stomach is much less typical than in the colon. Typically, the adenomatous polyps, which are rather seldom, are the polyps with the highest malignant potential. To be complete, it has to be added that ECL cell-derived neuroendocrine tumors (NETs) are associated with both Hp infectious gastritis (43) and autoimmune gastritis (44). Mutation in the gene CDH1 coding for E-cadherin predisposes to gastric carcinoma of diffuse type (45), and quite recently missense mutation of one of the genes of the proton pump, resulting in anacidity from birth was reported to give ECL cell-derived NETs of different degree of malignancy at an early age (46, 47).

## Classification of Gastric Carcinomas

Quite recently, a molecular classification of gastric carcinomas based on occurrence of mutations was described (48). Although such a system may have impact on the choice of treatment at the time of classification, it may not tell much about the cell of origin, which is mandatory for understanding of carcinogenesis and prophylaxis of tumors. In that respect, older histological classifications may be more useful.

**Figure 1:**
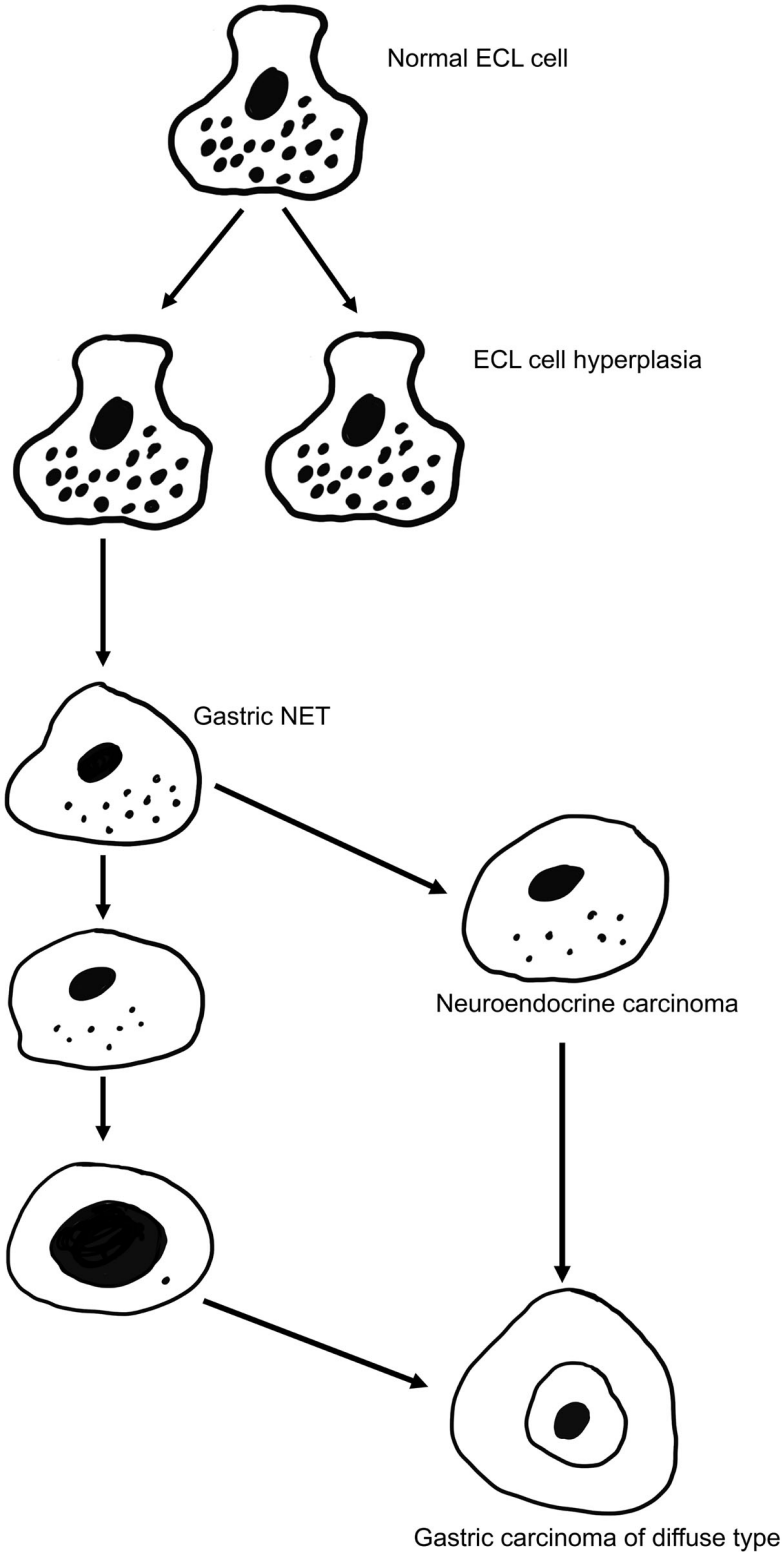
**The gradual transition of an enterochromaffin-like (ECL) cell into a highly malignant carcinoma cell of gastric carcinoma of diffuse type [from Waldum et al. (57) with permission]**.

There are many classification systems for gastric carcinomas, but the system proposed by Lauren seems to be the most relevant (49). Thus, his classification into intestinal and diffuse types where the intestinal type shows glandular structures, whereas the diffuse type lacks such differentiation is increasingly used most likely because the carcinomas do not transform into each other over time (49). In fact, the stable phenotype of the carcinomas during the course of the disease may suggest important biological differences that are supported by different epidemiology for the types of cancer (50). The main weakness with Lauren’s classification is that about 15% of the carcinomas are difficult to classify due to overlapping traits (49). Although gastric carcinomas of diffuse type lack a hallmark of adenocarcinomas (glandular growth pattern), they have nevertheless been classified as adenocarcinomas due to presumed mucin content of the cancer cells (49). The presence of mucin has been based on PAS positivity. However, PAS positivity is an unspecific histochemical method reacting with glycoproteins/peptides in general (51), and we have shown that the diffuse gastric carcinomas with the highest content of PAS positive material, the signet ring cells, are positive for NE markers both by immunohistochemistry (52) and *in situ* hybridization (53). In general, gastric carcinomas of diffuse type often show NE differentiation (54, 55), and it might be that this type actually is a NE carcinoma (Figure 1). In this context, it should be recalled that it has for long been known that NETs (formerly called carcinoids) can show glandular growth pattern (56, 57) and mucin positivity (58). Since the gastric carcinomas with endocrine differentiation (which mainly are of diffuse type), more specifically seem to be of ECL cell origin (59), the principal regulator of ECL cell function as well as growth, gastrin, naturally becomes of central interest in gastric carcinogenesis.

## Gastrin and Gastric NETs

More than 30 years ago, gastrin was realized to induce gastric NETs in man whether due to gastrinoma with increased gastric acid secretion (60) or being secondary to hypoacidity (61) causing Bordi to raise the question whether this was a hormone induced tumor (62). These tumors were regarded as rather benign and also very rare. Gastric NETs were therefore not seen as very important from a clinical point of view. With the description of NETs in the oxyntic mucosa of rodents dosed long term with inhibitors of gastric acid secretion belonging to the histamine receptor-2 blockers (H-2 blockers) like loxtidine (63) or the proton pump inhibitors (PPIs) omeprazole (64), the interest increased dramatically since these drug were so commonly used. It was soon realized that hypergastrinemia was the common pathogenic factor and that the degree and duration of hypoacidity and secondary hypergastrinemia determine the risk of tumor development. Thus, although very high doses of the commonly used H-2 blockers like ranitidine can induce such tumors in rodents (65), the doses used clinically carry little risk since the potency of H-2 blockers in clinical use is too low. Moreover, the tolerance ordinary H-2 blockers induce (66) also protects against long-term marked hypoacidity/hypergastrinemia. Loxtidine was the so-called unsurmountable H-2 blocker (63), and this compound was not developed into a drug due to the NETs seen in rodents. The PPIs are very efficient drugs in inhibiting gastric acid secretion (67), but at the same time risky due to hypergastrinemia. Rather early, it was shown that PPI treatment induced ECL cell hyperplasia (68), which caused rebound acid hypersecretion (69). A decade later, it was reported that a period of PPI dosed to healthy individuals induced dyspepsia (70) probably due to rebound acid hypersecretion. Studies based on treatment of PPIs for up to 10 years have not reported occurrence of gastric NETs (71, 72), and thus such treatment has therefore been claimed to be safe with regard to neoplasia. However, it has to be remembered that neoplasia and particularly with respect to tumors originating from NE cells, which are so slow proliferators, may take decades to develop. Moreover, already more than 15 years ago, the producer reported on the occurrence of reversible ECL cell NETs during omeprazole treatment (73), and we (74) and others (75, 76) have reported similar tumors during PPI treatment. Finally, and very importantly, a large kindred from Spain was reported to develop ECL cell tumors of different degree of malignancy at a young age (20s and 30s) due to missense mutation causing lack of function of the proton pump. Thus, the homozygote individuals who all developed tumors had been anacidic and hypergastrinemic from birth (46). Most of the tumors were classified as NETs although one tumor was diagnosed as an adenocarcinoma (46). Later, we have examined these tumors and have concluded that the adenocarcinoma more correctly should have been classified as a NE neoplasm (47). We have also described a gastric NE carcinoma that developed after about 12 years of PPI treatment (77). Oxyntic gastric neoplasia has been reported in all conditions with hypergastrinemia in animals as well as man. It is therefore no doubt that hypergastrinemia may lead to gastric NETs. The dominating role of gastrin in the pathogenesis of gastric NETs is demonstrated by their eradication by the treatment with the gastrin antagonist netazepide (78). We have also demonstrated that treatment with a long-acting somatostatin analog makes gastric NETs disappear or shrink (79). This effect was most probably due to a direct effect on the tumor cells since the reduction in blood gastrin was only marginal (79). On the other hand, patients operated with antrectomi due to peptic ulcer either as Billroth-I or particularly Billroth-II do develop after long latency carcinomas in the remnant stomach even though they are hypogastrinemic (80). However, not only the ECL cell but also other NE cells in the gastric mucosa could possibly give rise to tumors. Thus, we have described a case where a cancer of the remnant stomach originating from the D cell, developed decades after the Billroth-II procedure (81). It has not yet been examined, but it is conceivable that gastrin has a negative trophic effect on the D cell in the oxyntic mucosa.

## Gastrin and Gastric Carcinomas

**Figure 2:**
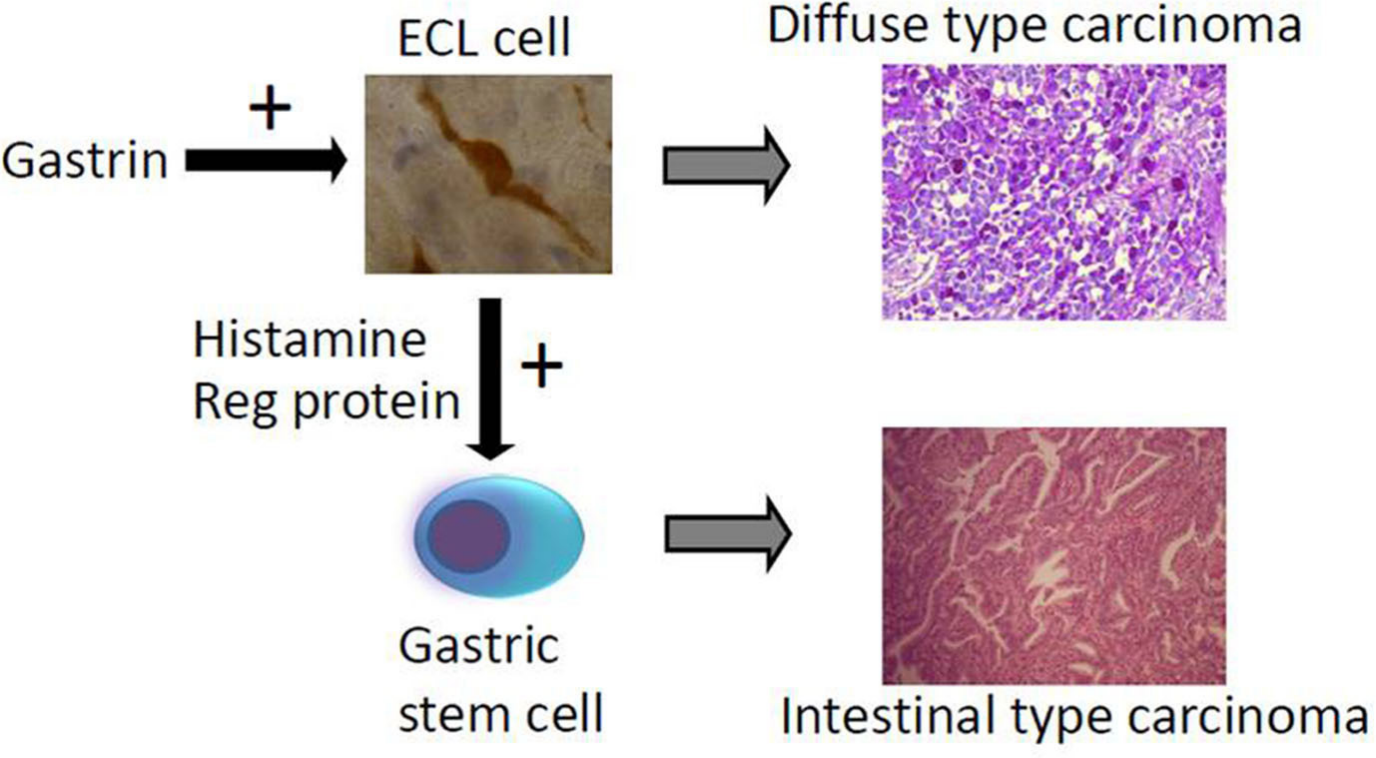
**The way hypergastrinemia may induce gastric carcinoma of both diffuse and intestinal types [from Waldum et al. (89) with permission]**.

The main cause of gastric carcinomas is Hp infection (31) and mainly when inducing atrophy of the oxyntic mucosa (36). Atrophic oxyntic gastritis leads to reduced acid secretion, gastric hypoacidity, and secondary hypergastrinemia (26). We have repeatedly shown that gastric carcinomas, especially of diffuse type, express NE markers (54, 55) and more specifically ECL cell markers (59), which incriminate gastrin in the pathogenesis. We have described a patient with pernicious anemia who developed a gastric NET that was removed endoscopically and after 1 year had recurrence of the tumor, resulting in gastrectomy and who died some years later of general metastasis (82). During the 5-year period after the first diagnosis of the gastric NET, the tumor showed progressive loss of NE markers and a dramatic increase in proliferation demonstrating the transition from a gastric NET into a highly malignant carcinoma (82). The close relationship between gastric NETs and gastric carcinomas is also reflected by the co-occurrence of NETs and carcinomas in autoimmune gastritis (83) as well as Hp gastritis predisposing both to gastric cancer (31) and gastric NETs (43). A few studies have reported hypergastrinemia in patients with gastric carcinoma (84–86). The gastric carcinomas of diffuse type quite possibly originate from the ECL cell (54, 55, 59) by dedifferentiation. Carcinomas of intestinal type are also associated to atrophic oxyntic gastritis (87), but this type of gastric carcinoma more seldom express NE markers and therefore more likely develop from the stem cells. However, gastrin may nevertheless be important in the carcinogenic process leading to carcinoma of intestinal type by stimulating the proliferation of the stem cell, either by a possible but not proven gastrin receptor on this cell or indirectly by a positive trophic effect of mediators from the ECL cell, among them Reg protein is the most likely candidate (88) (Figure 2).

Since the acknowledgment of the central role of Hp infection in gastric carcinogenesis (31), there has been an unsuccessful search for the mechanism. However, it seems logical to focus on the gastritis since also the so-called autoimmune gastritis predisposes to gastric cancer (33). Moreover, oxyntic gastric atrophy is a common feature in conditions causing gastric cancer (36), and therefore, we recently proposed that hypergastrinemia could be the factor incriminated in Hp gastric carcinogenesis (89). The risk of long-term profound inhibition of gastric acid secretion particularly by the very efficient PPIs is increasingly recognized (90, 91). To conclude this part, gastrin seems to be the most important factor in gastric carcinogenesis.

## Consequences of the Dominating Role of Gastrin in Gastric Carcinogenesis

The understanding of the fundamental role of gastrin in gastric carcinogenesis is of clinical importance. Thus, we should avoid long-term treatment particularly in children and young adults due to their long life expectancy, with inhibitors of gastric secretion leading to hypergastrinemia. Presently, long-term treatment with PPI is mainly indicated in patients with gastroesophageal reflux disease (GERD). In young GERD patients, H-2 blocker treatment should be the first choice in order to reach the goals (acceptable symptomatic relief and healing of lesions) at the lowest level of acid inhibition as possible. The alternative of anti-reflux surgery must also be carefully evaluated in these patients. When PPI treatment is applied, gastrin and chromogranin A, which in this setting are good markers of the ECL cell mass (22, 69, 92), should be monitored. It may in the future be possible to treat young patients with oxyntic atrophic gastritis and hypergastrinemia with a gastrin antagonist as prophylaxis against cancer. Furthermore, at which stage of tumor development gastrin loses its effect must be elucidated, in order to determine whether a gastrin antagonist could be used in the treatment. Indirectly, this may be assessed by examination of the tumor cells for the presence of the gastrin receptor either by immunohistochemistry or by *in situ* hybridization.

## Conclusion

Gastrin and its target cell, the ECL cell, probably play a central role in gastric carcinogenesis.

## Author Contributions

HW has been the leader of this research throughout many decades. LS has participated in the work during the last years making a thesis on the subject. She has participated active in making the manuscript. PM is a pathologist presently making her doctoral degree on the classification of gastric and renal carcinomas. She has done the evaluation of tumor tissue during the last years and has also participated in the writing of the manuscript.

## Conflict of Interest Statement

The research behind this publication has been conducted in the absence of any commercial or financial relationship that could be construed as a potential conflict of interest.
